# Removal of Colon for Obstructive Faecal Stasis

**Published:** 1914-09

**Authors:** 


**Removal of Colon for Obstructive Faecal Stasis.** John G. Clark, Philadelphia, Penn. Med. Jour., April, 1914, reports 8 cases. He calls attention to the fact that the colon is, more than any other organ, subject to normal variations in growth and rotation, so that no measurement nor topographic marking nor X-Ray picture alone can determine whether it is normal or not. The diagnosis must be made by definite and characteristic symptoms, the X-Ray being of value to determine whether stasis exists or not. In 5 cases radical relief of constipation resulted; thirst, to be expected from theoretic physiologic considerations existed only in one case and then not to an immoderate degree; equally contrary to theory, post-operative diarrhoea did not occur in 7 cases and developed only after several months in the single case; 5 cases gained considerably in weight; striking general improvement occurred in 3 cases, it was partial and doubtful in 2, there was complete failure in 2, and in one death occurred after some months from post-operative adhesions. (Note: The fact that a moderate sagging of the transverse colon in an X-Ray picture should not be considered an indication of ptosis and for colopexy, is one that we have insisted on for some time. Somehow, conceptions of intestinal locations and forms have developed without justification and have persisted in spite of ample post mortem information as to very variable positions. The term sigmoid is a good example. This part of the bowel assumes a great variety of shapes but we do not remember ever to have seen it in the form of either a sigma or the Roman S.—Ed.)

